# The *C. elegans Hox *gene *ceh-13 *regulates cell migration and fusion in a non-colinear way. Implications for the early evolution of *Hox *clusters

**DOI:** 10.1186/1471-213X-10-78

**Published:** 2010-07-28

**Authors:** Borbála Tihanyi, Tibor Vellai, Ágnes Regős, Eszter Ari, Fritz Müller, Krisztina Takács-Vellai

**Affiliations:** 1 Department of Genetics, Eötvös Loránd University, Budapest, H-1117, Hungary; 2 Department of Biology, University of Fribourg, Fribourg, CH-1700, Switzerland

## Abstract

**Background:**

*Hox *genes play a central role in axial patterning during animal development. They are clustered in the genome and specify cell fate in sequential domains along the anteroposterior (A-P) body axis in a conserved order that is co-linear with their relative genomic position. In the soil worm *Caenorhabditis elegans*, this striking rule of co-linearity is broken by the anterior *Hox *gene *ceh-13*, which is located between the two middle *Hox *paralogs, *lin-39 *and *mab-5*, within the loosely organized nematode *Hox *cluster. Despite its evolutionary and developmental significance, the functional consequence of this unusual genomic organization remains unresolved.

**Results:**

In this study we have investigated the role of *ceh-13 *in different developmental processes, and found that its expression and function are not restricted to the anterior body part. We show that *ceh-13 *affects cell migration and fusion as well as tissue patterning in the middle and posterior body regions too. These data reveal novel roles for *ceh-13 *in developmental processes known to be under the control of middle *Hox *paralogs. Consistently, enhanced activity of *lin-39 *and *mab-5 *can suppress developmental arrest and morphologic malformation in *ceh-13 *deficient animals.

**Conclusion:**

Our findings presented here show that, unlike other *Hox *genes in *C. elegans *which display region-specific accumulation and function along the A-P axis, the expression and functional domain of the anterior *Hox *paralog *ceh-13 *extends beyond the anterior region of the worm. Furthermore, *ceh-13 *and the middle *Hox *paralogs share several developmental functions. Together, these results suggest the emergence of the middle-group *Hox *genes from a *ceh-13*-like primordial *Hox *ancestor.

## Background

**Figure 1:**
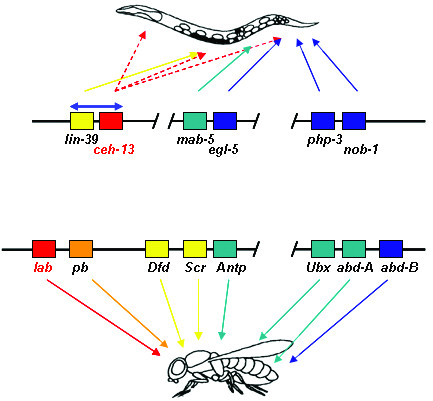
**The *C. elegans *and *Drosophila Hox *clusters**. The orthologs are indicated by the same coloring. Arrows point to body domains where *Hox *genes exert their action. Dotted red arrows indicate body parts in which *ceh-13 *is expressed. The blue double arrow indicates the inversion event that occurred between the ancestors of *ceh-13 *and *lin-39*. Color meanings: red and orange, anterior paralogs; yellow and green, middle paralogs; dark blue, posterior paralogs.

One of the most striking shared developmental mechanisms in divergent animal phyla is the patterning of the anteroposterior body axis by evolutionarily conserved homeodomain-containing transcription factors encoded by *Hox *genes [1-5]. Properties of the *Hox *genes include clustering in the genome, a conserved order within the cluster, a co-linear arrangement of their genomic position and functional domain in the body, and a hierarchy of action between the adjacent *Hox *paralogs. In *C. elegans*, the *Hox *cluster consists of six *Hox *genes that represent three paralogous groups: one anterior *Hox *gene, *ceh-13 *(C. elegans *homeobox-containing gene*), two middle-group paralogs, *lin-39 *(*lineage defective*) and *mab-5 *(*male abnormal*), and three posterior paralogs, *egl-5 *(*egg-laying defective*), *nob-1 *(*no backside*) and *php-3 *(*posterior *Hox *gene paralog*) (Figure 1) [6-14].

The *C. elegans Hox *genes differ in some characteristics from their counterparts in other animal phyla. For example, embryonic patterning and viability in *C. elegans *require only the anterior and the two most posterior *Hox *genes; triple loss-of-function (lf) mutant worms defective for *lin-39*, *mab-5 *and *egl-5 *can develop into fertile adults [3,15], raising interesting questions about the evolutionary status of these dispensable *Hox *genes. They control various developmental processes, including the migration of Q neuroblasts, cell fusion in Pn.p cell lineages, cell fate specification during vulval patterning, and programmed cell death [11,12,16-18].

Another unique feature of the *C. elegans Hox *genes is the unusual position of the anterior *Hox *paralog *ceh-13*, the nematode counterpart of *Drosophila labial *and mammalian *HoxB1 *[9,13,14]. *ceh-13 *is located downstream of the middle *Hox *gene *lin-39*, the worm ortholog of *Drosophila Deformed/Sex comb reduced *and mammalian *HoxD4 *(Figure 1), thereby representing a break in co-linearity. The functional consequence of this unusual genomic organization and the role of *ceh-13 *in development remain largely unknown.

*sw1*, a strong lf mutation in *ceh-13*, disrupts normal patterning of the anterior body part and arrests development during embryonic or early larval stages [14]. However, a small percent of *ceh-13(sw1) *mutants are able to develop into fertile adults, suggesting that this gene shares developmental roles with (an)other *Hox *paralog(s). Regardless of the anterior manifestation of the pleiotropic Ceh-13 mutant phenotype, *ceh-13 *displays a complex, highly dynamic expression pattern, which involves several different cell lineages all over the body, even in the developing tail region [13,19,20]. These data raise the intriguing possibility that the influence of *ceh-13 *on cell fate specification may not be restricted to the anterior body part, and its functional domain may overlap with that of other *Hox *paralogs.

The proper function of HOX proteins requires TALE homeodomain proteins [21,22]. In *C. elegans*, these HOX cofactors are encoded by *ceh-20 *and *unc-62 *(*uncoordinated*), the orthologs of Extradenticle/Pbx and Homothorax/Meis/Prep genes, respectively [23-26]. Interestingly, the function of CEH-20 and UNC-62 is partly independent of LIN-39 and MAB-5 in regulating cell migration and fusion as well as vulval cell fate specification in the mid- and posterior body regions [23,25,26]. This indicates a potential role for (an)other *Hox *gene(s) in the control of these developmental processes.

In this study we have implicated a role for *ceh-13 *in positioning Q neuroblasts and the fusion process of Pn.p cells. Unexpectedly, the function of *ceh-13 *in these paradigms was obvious along the entire anteroposterior body axis. In addition, vulva patterning also appeared to be affected in *ceh-13 *mutant animals. Consistently, we found that the expression domain of *ceh-13 *overlaps with those of the other *Hox *paralogs, and *ceh-13 *interacts with *lin-39 *and *mab-5 *as the elevated levels of which are able to suppress the embryonic and early larval lethal phenotype of *ceh-13 *lf mutants. These findings suggest that in the nematode lineage the middle *Hox *genes emerged from a primordial *ceh-13*-like *Hox *paralog, and that the ancestor of this anterior *Hox *paralog might have given raise the primitive *Hox *cluster through tandem gene duplications during an early phase of animal evolution.

## Results

### *ceh-13 *is required for the positioning of Q neuroblasts

**Figure 2:**
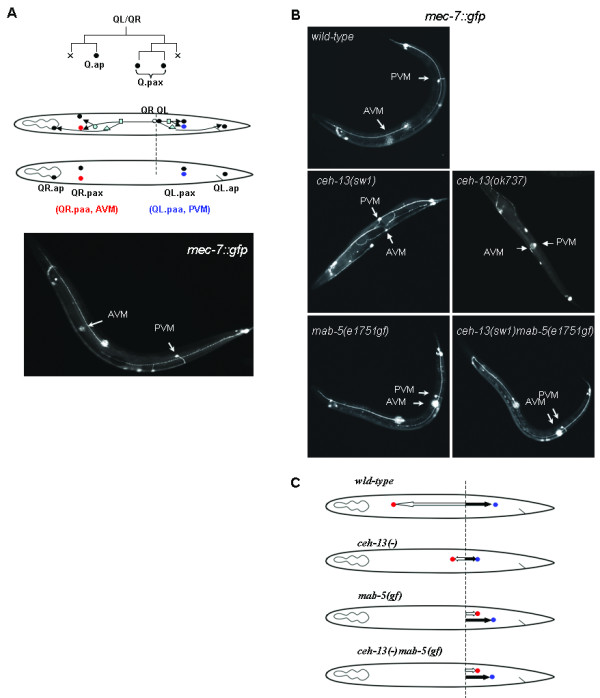
***ceh-13 *deficiency causes defects in cell migration**. **A**, The wild-type Q cell lineage (top) and migration pattern (middle). The two Q daughters, QL and QR, generate identical cell lineages to produce three neurons (circles). "x" indicates apoptotic cell death. The bottom panel shows the final position of the Q cell descendants. AVM and PVM are indicated by red and blue circles, respectively. *mec-7 *is expressed in these two cells within the Q lineage. The fluorescence image shows the expression of a *mec-7::gfp *reporter in wild-type background. AVM and PVM are indicated. **B**, *mec-7::gfp *expression in a wild-type, *ceh-13(-) *single mutant, *mab-5(gf) *single mutant and *ceh-13(-)mab-5(gf) *double mutant animal. The positions of AVM and PVM are indicated. **C**, Schemes showing the migration pattern of two Q neuroblast descendants, AVM and PVM, in *ceh-13(-) *and *mab-5(gf) *single mutants, as well as in *ceh-13(-)mab-5(gf) *double mutant background.

Positioning of Q cell descendants provides an excellent paradigm to study how *Hox *genes affect cell migration during development. The QR and QL neuroblasts are born by the division of the Q cell in the posterior body part of the animal, and initially located directly opposite to each other. During the early larval stages, QR and then its descendants migrate toward the anterior, while QL and its descendants migrate posteriorly (Figure 2A) [27]*lin-39 *and *mab-5 *control the migration of these cells in a concerted manner [11,12]. The migration of QR and its descendants requires *lin-39 *activity: *lin-39 *deficiency blocks the migration of these cells prematurely at various positions. The migration of QL and its descendants is influenced by *mab-5*. Inactivation of *mab-5 *renders these cells to be incapable of migrating posteriorly; instead, they migrate toward the head. Conversely, a gain-of-function (gf) allele of *mab-5*, *e1751*, causes QR, which normally moves anteriorly, to migrate toward the tail (Figure 2B, C) [11,12]. The migration of Q neuroblasts is also influenced by *ceh-20 *and *unc-62*, which are predicted to function as cofactors of *lin-39 *and *mab-5 *in these processes. However, *lin-39(-)ceh-20(-) *and *mab-5(-)ceh-20(-) *double mutant animals exhibit more severe defects in the migration of these cells than either of these single *Hox *mutants [25]. This suggests a role for CEH-20 in this cell migration paradigm which is independent of LIN-39 and MAB-5.

**Figure 3:**
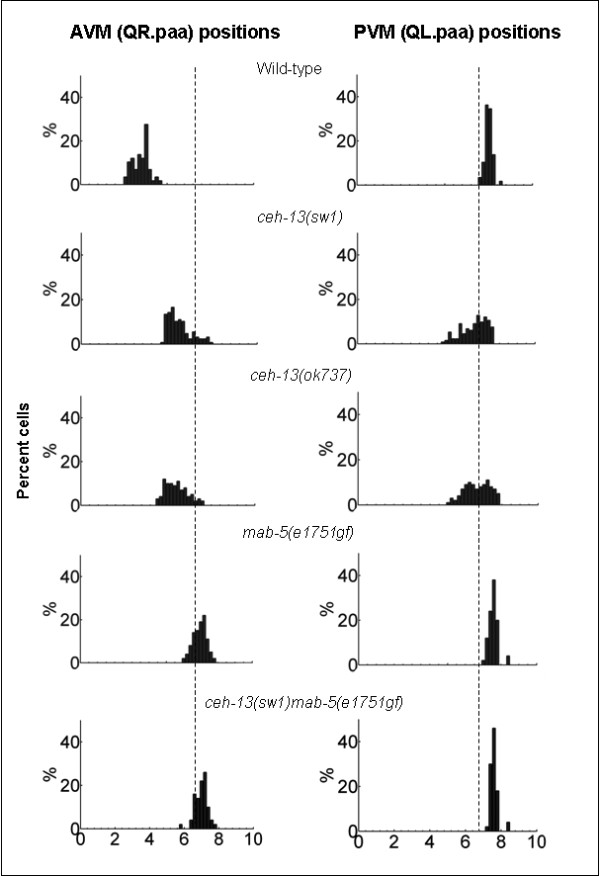
**Cell migration defects in *ceh-13(-) *mutant animals**. Relative final positions of AVM and PVM are indicated by bars. The length of the X axis corresponds to the relative length of the animals. The left is at anterior. The vertical dotted lines indicate the birthplace of the Q cells. 80 animals were scored for each strain. When PVM does not migrate or migrates anteriorly relative to its birthplace, it is considered as mutant for migration behavior.

To examine whether CEH-13 influences cell migration in the Q cell lineage, we monitored the final position of two Q descendants, QR.paa (AVM) and QL.paa (PVM), in wild-type versus *ceh-13 *lf mutant background. To visualize these cells, we used an integrated green fluorescent protein- (GFP) labeled *mec-7 *reporter [28], which is expressed only in these two neurons within the Q lineage (Figure 2A). We found that in *ceh-13(sw1) *mutant animals, AVM stops to migrate toward the anterior prematurely (Figs. 2B, C and 3). As a result, AVM was often located close to the central body region. This mutant phenotype was expressed with almost a full penetrance in *ceh-13(sw1) *mutant larvae. A similar Q cell lineage-specific migration defect was observed in *ceh-13(ok737) *mutants too (Figure 2B). We next assayed cell migration in the QL lineage in *ceh-13 *deficient background. To our surprise, defects in QL lineage positioning were also evident in *ceh-13(sw1) *mutant animals: PVM was located improperly in 22% (19 out of 86) of the animals examined (Figs. 2B, C and 3). In the affected larvae, this cell was unable to migrate to its normal final position. Although this migration defect was partially penetrant, we conclude that the functional domain of *ceh-13 *in controlling Q cell migration overlaps with that of *lin-39 *and *mab-5*.

The *mab-5(e1751gf) *mutation reverses the direction of AVM migration, but does not affect PVM migration [11,12]. We also analyzed the position of Q cell descendants in *ceh-13(sw1)mab-5(e1751gf) *double mutant animals, and found that AVM is located slightly posterior to the position where QL and QR are normally born (Figs. 2B, C and 3). Thus, *ceh-13 *and *mab-5 *may have opposite effects on this particular cell migration event, and the combination of the two mutations may inhibit the ability of AVM and its progenitors to migrate to their normal position.

**Figure 4:**
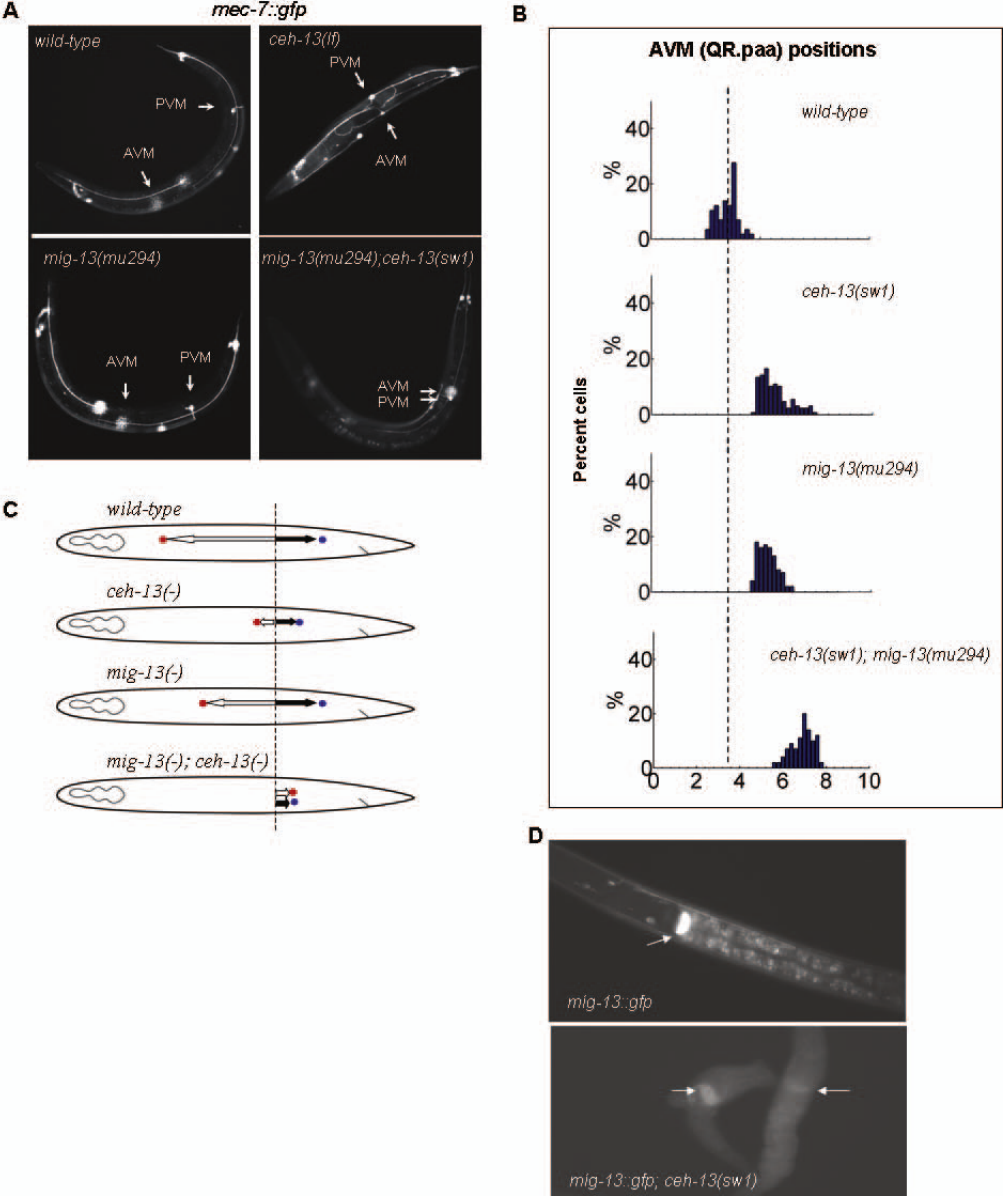
***mig-13 *and *ceh-13 *interact in controlling Q cell migration**. **A**, Fluorescent images showing *mec-7::gfp *expression in *ceh-13(-) *and *mig-13(-) *single mutant animal, as well as in a *ceh-13(-); mig-13(-) *double mutant animal. The positions of AVM and PVM are indicated by white arrows. **B**, Relative final positions of AVM in *mig-13 *and *ceh-13 *deficient animals. The length of the X axis corresponds to the relative length of the animals. The left is at anterior. Vertical dotted lines indicate the birthplace of the Q cells. At least 100 animals were scored for each strain. **C**, Schemes showing the migration pattern of AVM and PVM in *ceh-13(-) *and *mig-13(-) *single mutant backgrounds, as well as in *ceh-13(-); mig-13(-) *double mutant background. **D**, *mig-13 *expression requires *ceh-13 *activity. White arrows point to the pharyngeal-intestinal valve cells. The vast majority of *ceh-13(-) *mutants failed to or weakly express *mig-13 *(93%, N = 133). Mutant animals were captured with the same or even a longer exposure time than was applied for the wild-type background.

Normal positioning of AVM also requires *mig-13 *(*cell migration abnormal*), which codes for a novel transmembrane receptor [29]. The expression of *mig-13 *is restricted to the anterior body part by the inhibition of *mab-5*: *mig-13 *is normally active in certain cells of the ventral nerve cord (VNC) in the anterior half of the L1 stage larvae, but becomes ectopically expressed in the posterior body part in *mab-5 *lf mutants [29]. Since *ceh-13 *is also expressed in the VNC at this stage [14], we asked whether *ceh-13 *interacts with *mig-13 *to control AVM migration. We found that AVM displays more severe positioning defects in *ceh-13(sw1); mig-13(mu294) *double mutant animals than in either of the single mutants (Figure 4A-C). In a portion of these double mutants, AVM was positioned even toward the posterior (Figure 4A-C). In good accordance with these results, *mig-13::gfp *expression was completely abolished in *ceh-13 *mutant larvae (data not shown). In some *ceh-13 *deficient larvae, even the pharyngeal-intestinal valve cells, which always show a strong *mig-13 *expression throughout all larval stages in wild-type background, failed to express *mig-13 *(Figure 4D). These data raise the possibility that *ceh-13 *may control the anterior migration of cells in the Q lineage via influencing *mig-13 *activity. In summary, we conclude that *ceh-13 *is required for the normal positioning of both QR and QL descendants. The regulation of cell migration in the posterior part of the body by the anterior-like *Hox *gene *ceh-13 *was somehow unexpected.

### *ceh-13 *regulates the fusion process of Pn.p cells in both anterior and posterior body parts

**Figure 5:**
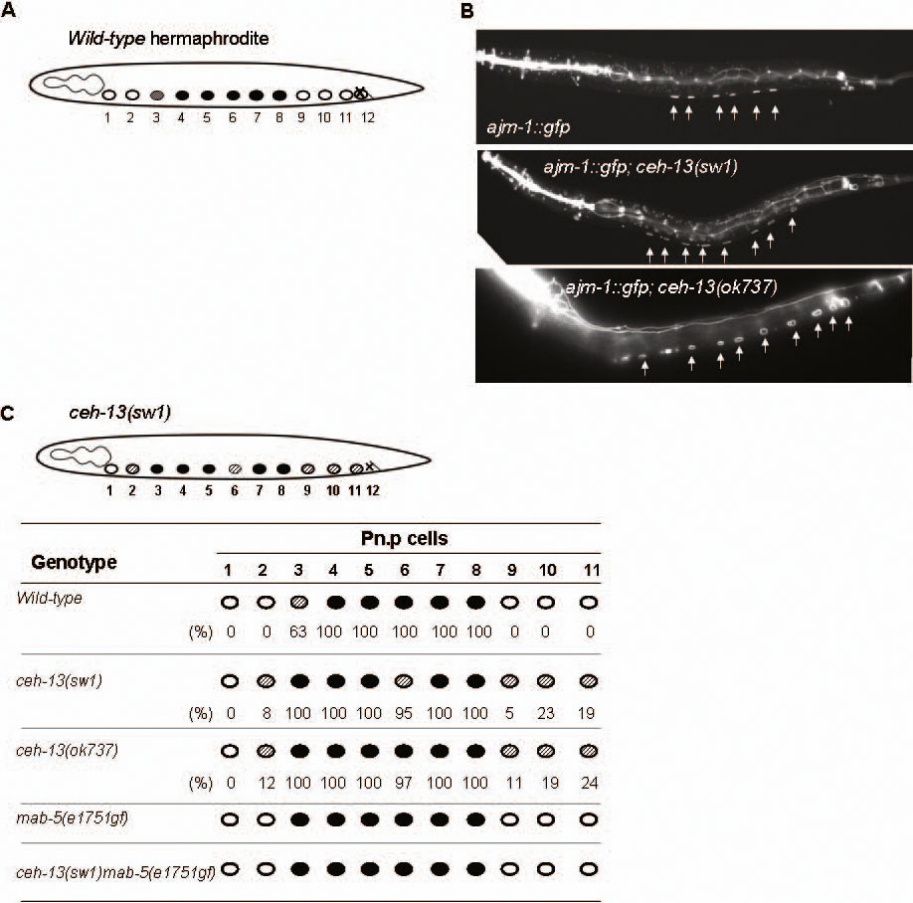
***ceh-13 *promotes cell fusion in Pn.p lineages**. **A**, Cell fusion pattern of the Pn.p cells in wild-type hermaphrodites at the L1 larval stage. Open circles represent Pn.p cells that fuse with the hypodermal syncytium hyp7, black circles represent Pn.p cells that remain unfused, shaded circle represents P3.p, which fuses with hyp7 only in nearly half of the animals. The Pn.p cells are indicated by numbering below the animal. **B**, An integrated *ajm-1::gfp *reporter outlines six unfused Pn.p cells, P(3-8).p, in a wild-type L2 stage larva (top), and, in addition to P(3-8).p, several other Pn.p cells that remain ectopically unfused in *ceh-13(-)*mutant L2 stage larvae (middle and bottom). The unfused Pn.p cells are indicated by white arrows. **C**, Effect of *ceh-13 *deficiency on the Pn.p cell fusion pattern. The fusion pattern in wild-type larvae, as well as in *ceh-13(lf) *and *mab-5(gf) *mutant larvae after the early fusion event at the L1/L2 larval stages (the numbers below the circles represents the percentage of the fusion events). 200 individual larvae were examined for each strain.

The fusion of certain epidermal blast cells with the hypodermal syncytium hyp7 represents another example, in which the distinct and combined regulatory functions of *lin-39 *and *mab-5 *have been well characterized [11,12]. At the early L1 larval stage, the ventral epidermis is composed of 12 ectodermal precursor cells, termed P(1-12), which are located in a row along the ventral surface of the animal (Figure 5A) [27]. At the late L1 stage, the P cells divide once, generating the Pn.a neuroblast and Pn.p epidermal daughters. Soon after their birth, some of the Pn.p cells fuse with hyp7, while the others remain unfused. The fusion pattern of Pn.p cells is established in a sex-specific manner, and regulated by *lin-39 *and *mab-5*. In wild-type hermaphrodites, the central P(3-8).p cells are prevented from undergoing fusion by *lin-39*. The fusion pattern of these cells is also influenced by CEH-20 [25]. In hermaphrodites defective for this HOX cofactor, P(3-8).p and some additional anterior and posterior Pn.p cells remain unfused. The fusion defective phenotype of *ceh-20 *mutants is obvious even in *lin-39 *null mutant background [25]. This is particularly interesting since in *lin-39 *single mutant animals each Pn.p cell fuses with hyp7 [11,12].

To examine whether *ceh-13 *is also involved in this cell fusion event, we scored the number of unfused Pn.p cells in *ceh-13 *mutant animals. The lack of the fusion process was identified by an integrated *ajm-1::gfp *(*apical junction molecule*) reporter that is specific for a component of adherens junctions and thus outlines unfused cells [30]. We found that in addition to the central P(3-8).p cells, several anterior and posterior Pn.p cells remain unfused in *ceh-13(sw1) *hermaphrodites: 8% of P2.p, 23% of P9.p and 19% of P10.p (N = 200) were unable to fuse with hyp7 in the mutant larvae examined (Figure 5B, C). *ceh-13(ok737) *mutants displayed similar defects in cell fusion (Figure 5B, C). Thus, the effect of *ceh-13 *on Pn.p fusion is not restricted to the anterior body part, but also extended to cells located in the posterior body region. The cell fusion defective phenotype of *ceh-13 *mutant larvae indicates that *ceh-13 *promotes the fusion of Pn.p cells with hyp7. We conclude that *ceh-13 *acts in an opposite way to LIN-39 to control this process. Interestingly, the fusion pattern of Pn.p cells in *ceh-13 *mutants highly resembles to that found in animals defective for *ceh-20 *[25]. These results suggest that *ceh-13 *is also involved in establishing the proper number of unfused Pn.p cells in hermaphrodites.

*mab-5 *does not affect Pn.p fusion in hermaphrodites [11,12]. However, the *mab-5 *gf mutation *e1751 *was able to restore the fusion defect of Pn.p cells in *ceh-13 *mutant background (Figure 5C). In the *ceh-13(sw1)mab-5(e1751gf) *double mutant animals that morphologically looked normal, the fusion pattern of the Pn.p cells appeared unaffected. Upon these results we suggest that ectopically or excessively expressed MAB-5 can substitute CEH-13 in cell fusion control.

### *ceh-13 *influences vulval patterning

In the *C. elegans *hermaphrodite, the vulval tissue - through which the animal lays embryos - develops from a subset of six Pn.p epidermal cells [P(3-8).p] called vulval precursor cells (VPCs), which lie ventrally along the anterior-posterior axis [27,31]. Although each VPC has the potential to adopt an induced vulval fate, normally only the three central VPCs, P(5-7).p, undergo vulval induction. The non-induced VPCs, P(3,4,8)p, divide once and their daughters fuse with hyp7. Descendants of the induced VPCs form eventually the matured vulval structure. The fate of VPCs is determined by the combined effect of multiple genetic cascades, including the Ras, Wnt and Notch signaling pathways, signaling via TRA-1 (sexual transformer) that is similar to the *Drosophila *Cubitus interruptus and mammalian GLI- (Glioma-associated) like proteins, and three redundant synMuv (for synthetic Multivulva) pathways grouped into classes A, B and C [31-34]. Under hyperinducing conditions, P(3,4,8).p can ectopically adopt induced vulval fates, which manifests in multiple vulval protrusions (Multivulva - Muv - phenotype). When vulval induction fails to occur, no VPC adopts vulval fate which renders the animal to exhibit a Vulvaless (Vul) phenotype.

**Figure 6:**
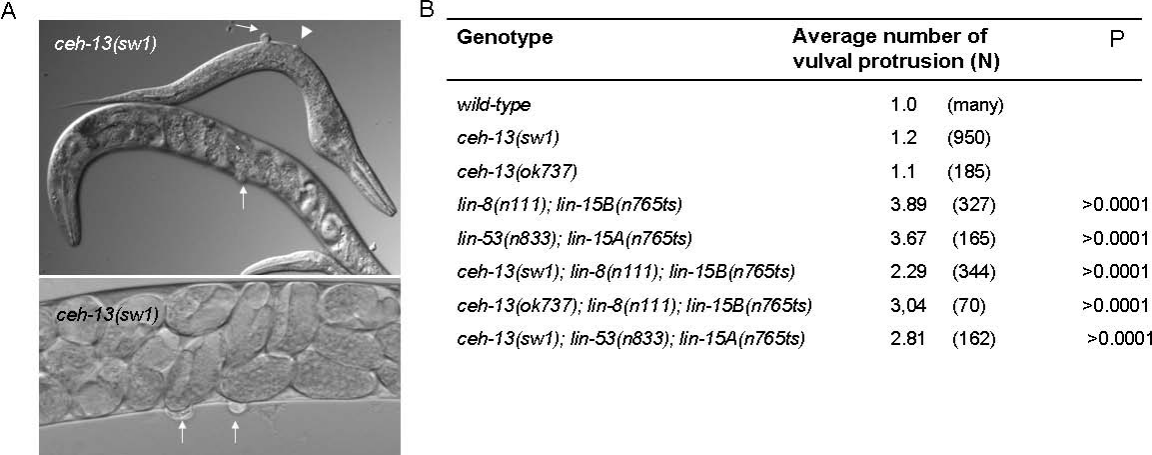
***ceh-13 *affects vulval patterning**. **A**, Vulval morphology in *ceh-13(-) *mutant hermaphrodites. White arrows indicate vulval protrusions (protruded vulval phenotype; Pvl), the arrowhead indicate an ectopic vulva (top). *ceh-13(-) *mutants exhibit variable vulval morphologies, including Pvl (8%), Multivulva (Muv; 0.2%) and Egg-laying defective (Egl; 14%) phenotypes (N = 200). **B**, Mutational inactivation of *ceh-13 *decreases vulval induction in *synMuv AB *double mutant animals. For each triple mutant: P < 0.001; for statistics, see the Material and Methods. *lin-8 *and *lin-15A *are class *A synMuv *genes, while *lin-53 *and *lin-15B *are class *B synMuv *genes. Single mutants defective in either of the synMuv A or B pathways have normal vulval morphology (data not shown), whereas *synMuv AB *double mutant are Muv. Wild-type animals actually have no vulval protrusion (their normal vulval structure is considered as 1 protrusion in the figure).

Both *lin-39 *and *mab-5 *affect vulval fate specification [34,35]. Furthermore, VPC induction in *ceh-20 *mutants can occur even in the complete absence of LIN-39 [25]. This knowledge prompted us to examine vulval morphology in *ceh-13 *mutant escapers. We found that these animals are Muv with a relatively low penetrance (<1%, N = 950). The affected adults had an extra vulval protrusion close to the normal vulva (Figure 6A). This phenotype is similar to that observed in *lin-39 *hypomorphic and *ceh-20 *lf mutants [26], suggesting that these factors, including *ceh-13*, have a similar role in VPC specification. To further assess this possibility, we monitored the effects of *ceh-13 *lf mutations on vulval induction in *synMuv AB *double mutant background. Single mutations in either of the SynMuv A or B pathway do not affect vulval development, whereas simultaneous inhibition of the two pathways renders the animals to be Muv [32]. Like *lin-39 *deficiency [36], inactivation of *ceh-13 *significantly suppressed vulval induction in *synMuv AB *double mutant background (Figure 6B). Thus, *ceh-13 *endorses VPCs to adopt an induced vulval fate (note that this complex vulval morphology of *ceh-13 *lf mutants - i.e., an extra vulval protrusion that is due to hypomorphic mutations and suppression of vulval induction in *synMuv AB *double mutant background by lf mutations - is also characteristic for *lin-39 *deficient animals [26]). It may exert this function by directly controlling some target "vulval" genes. Alternatively, the absence of *ceh-13 *activity may alter the expression domain of the middle *Hox *paralogs, especially that of *lin-39*, thereby influencing vulval patterning. Nevertheless, *ceh-13 *has obvious developmental roles in the mid-body region as well.

### The expression domain of *ceh-13 *overlaps with that of the other *Hox *paralogs

**Figure 7:**
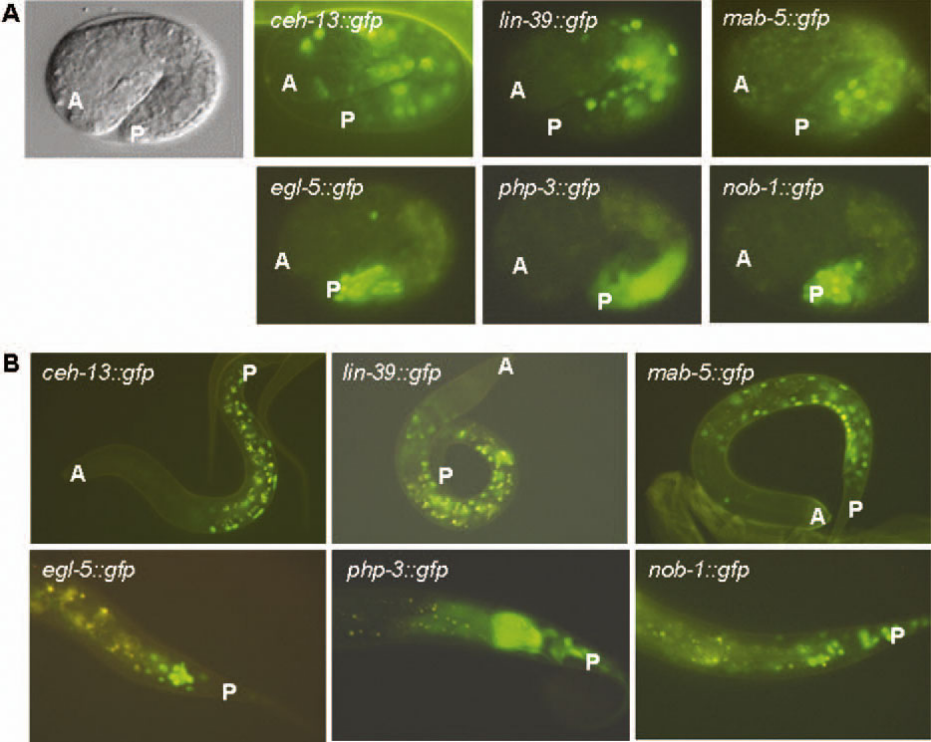
**The expression domain of *ceh-13 *overlaps with that of the other *Hox *paralogs**. **A**, Fluorescence images showing the expression of the *C. elegans Hox *genes at the two-fold embryonic stage. *ceh-13 *expression runs all along the anteroposterior body axis, ranging from head-to-tail. A: anterior, P: posterior. **B**, Expression of the *C. elegans Hox *genes at the L1 larval stage. Specimens transgenic for a posterior *Hox *paralog are shown only at the tail region (the bottom panels). A: anterior, P: posterior.

Although *ceh-13 *is most similar to the anterior *Hox *orthologs, e.g., *Drosophila labial *and mammalian *HoxB1*, its functional domain is obvious all along the anteroposterior body axis (see results above). Indeed, a transcriptional fusion *ceh-13::gfp *reporter, pMF1, has been previously reported to be expressed in many different cell lineages even from the mid- and posterior body parts during development [20,13,14]. To understand better this unusual - i.e., non-colinear - activity domain of *ceh-13*, we examined its expression in relation to that of the other *Hox *paralogs (Figure 7). To this end, we first generated *gfp*-labeled translational fusion reporters for *C. elegans Hox *genes whose expression has not been determined by such a system (see the Methods). The reporters we generated contain at least 9-10 kb large upstream regulatory sequences and almost the entire coding regions, and were able to partially rescue the corresponding mutant phenotype (data not shown). In two-fold stage embryos, *nob-1*, *php-3 *and *egl-5 *each were expressed in the tail region, as expected or previously reported (Figure 7A). Therefore, these posterior *Hox *paralogs, similar to their *Drosophila *and mammalian orthologs, are active in the tail region exclusively. In good agreement with previous results [8,12], LIN-39 accumulated in the mid- and posterior body region, whereas *mab-5 *was expressed in the posterior half of the body at the two-fold embryonic stage (Figure 7A). The expression of *ceh-13*, however, was evident in each of the main body domains, which is consistent with its functional properties. Based on these results, we suggest that *ceh-13 *is a unique *C. elegans Hox *gene in that it is expressed and functions in a non-colinear way. Its expression domain overlaps with those of the other *Hox *paralogs.

We also examined the expression of the *C. elegans Hox *genes at the L1 larval stage, and found a similar pattern that characterizes them during embryonic development: the posterior *Hox *paralogs were active in the tail, LIN-39 was expressed mainly in the mid-body region, the expression of *mab-5 *was restricted to the domain located between the posterior and central body parts, whereas *ceh-13 *expression was apparent along the entire anteroposterior body axis (Figure 7B). *ceh-13 *expression, for example, was detectable in nearly each of the P blast cells (Figure 7B) and their daughters [13]. Different neuronal precursors as well as lateral hypodermal cells were also CEH-13::GFP-positive in each of the main body regions. Thus, the expression domain of *ceh-13 *may have been extended from the anterior during evolution, presumably following the reciprocal translocation of *ceh-13 *and its closest *Hox *paralog, *lin-39*.

### Extra copies of *lin-39 *and *mab-5 *can suppress the pleiotropic Ceh-13 mutant phenotype

**Figure 8:**
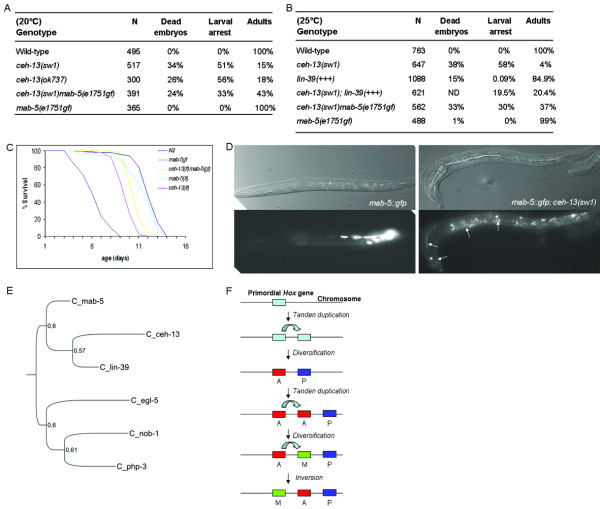
**Increased activity of *lin-39 *and *mab-5 *can rescue some defects in *ceh-13 *mutant animals**. **A-B**, A gain-of-function mutation in *mab-5*, *e1751*, suppresses lethality in *ceh-13(-) *mutants. Note that most of the double mutant animals look superficially normal. **B**, Extra copies of *lin-39 *suppress larval lethality in *ceh-13(sw1) *mutants. "+++" represents an integrated translational fusion LIN-39::GFP reporter that is able to rescue the Vul phenotype of *lin-39 *hypomorph mutants (~5%, N = 122). ND, not determined. **C**, *mab-5(e1751gf) *mutation restores the lifespan of *ceh-13(-) *mutants to a nearly normal level. Note that the vast majority of *ceh-13(-) *single mutants die at early stages of development (Figure 8A, B), and the survivals (escapers) live only few days as adults (i.e., they are extremely short-lived). For double mutants, normal looking L4 stage larvae were selected and then scored for survival (yellow curve). Samples were assayed in triplicates. For *ceh-13(sw1) *single mutants, ~70 mutant escapers were examined in each assay. For the other strains, 150-150 animals were scored in each assay. p < 0.001, when *ceh-13(lf)mab-5(gf) *double mutants were calculated to *ceh-13(lf) *single mutants by pair-wise comparisons, and p > 0.5 when double mutants were compared to the wild type. The log-rank test was used for comparison. N2 indicates wild-type. **D**, *mab-5::gfp *expression in wild-type (left panel) vs. *ceh-13(sw1) *mutant (right panel) background at the L1 larval stage. Nomarski pictures on the top, corresponding fluorescent images at the bottom. White arrows indicate cells that are gfp-positive. **E**, Phylogenetic tree of homeodomain sequences of the *C. elegans Hox *genes. The tree was generated by the Bayesian phylogenetic method. Numbers at the nodes correspond to the probability - i.e., clade credibility - of each node (from 0 to 1). **F**, Model for the early evolution of the nematode *Hox *cluster. A *ceh-13*-like primordial *Hox *gene underwent a tandem gene duplication event in an early phase of animal evolution, leading to the ancestors of the primitive anterior and posterior *Hox *genes. A subsequent tandem duplication of the anterior ancestor resulted in the ancestor of the middle-group *Hox *genes. A: anterior, P: posterior, M: Middle.

*ceh-13 *single mutant animals that are able to develop into adulthood are small, exhibit a variable abnormal morphology and reduced fertility, and have a slow growth rate [14]. As the expression domain of *ceh-13 *overlaps with those of the other *C. elegans Hox *paralogs, one might expect functional redundancy between the anterior *Hox *gene and other *Hox *paralogs. Indeed, we found that a translational fusion LIN-39::GFP reporter, *zhIs1*, is able to rescue larval, but not embryonic, lethality of *ceh-13 *mutants (note that this transgene was able to rescue the Vul phenotype of *lin-39 *hypomorph mutants). Whereas less than 4% (13/437) of the *ceh-13(sw1) *mutant larvae developed into fertile adults only, a large portion of *ceh-13 *mutants bearing *zhIs1 *were able to pass the larval stages (Figure 8A, B). In addition, the growth rate of *ceh-13 *mutants was significantly faster with the reporter than without the reporter (data not shown).

We also used a *mab-5 *gf mutation to test functional redundancy between *ceh-13 *and this middle *Hox *paralog. We found that the majority of *ceh-13(sw1)mab-5(e1751gf) *double mutants develop normally (Figure 8A, B). The morphology and behavior of these animals appeared to be superficially wild-type. Consistently, the lifespan of *ceh-13(sw1)mab-5(e1751gf) *double mutant animals were also comparable with that of the wild type (Figure 8C). Thus, increased dosage of *lin-39 *and *mab-5 *may substitute *ceh-13 *function in certain developmental processes. This can explain why half of the mutants defective for *ceh-13 *are able to pass embryonic development, the existence of *ceh-13 *escapers, as well as the viability of *lin-39mab-5 *lf double mutants.

We also considered the possibility that *ceh-13 *is involved in regulating the expression of *lin-39 *or *mab-5 *in certain cell types. To address this issue we monitored *mab-5 *activity in *ceh-13(sw1) *mutant background, and found significant changes in the expression of *mab-5*, as compared with the wild-type background. For example, at the L1 larval stage *mab-5 *was ectopically expressed in the head, while its expression was strongly reduced in the tail region of *ceh-13 *mutants (Figure 8D). In contrast, *lin-39 *expression was hardly changed in *ceh-13 *mutants. We conclude that a complex - region- and stage-specific - regulatory relationship exists between *ceh-13 *and the middle *Hox *paralogs.

Finally, we performed a Bayesian phylogenetic analysis of the *C. elegans Hox *homeodomain sequences. On the tree generated the middle paralogs stem from the anterior *Hox *branch, further supporting their close relation to *ceh-13 *(Figure 8E). In the light of these finding, the role of *ceh-13 *in controlling the migration of posterior Q cell descendants, fusion of posterior Pn.p cells, and vulval induction is now better understandable. Together, these data provide a functional support for the common evolutionarily origin of *ceh-13 *and the middle *Hox *paralogs (Figure 8F).

## Discussion

Genes from the *Hox *gene clusters encode evolutionarily conserved homeodomain-containing transcription factors that play a pivotal role in axial patterning during animal development [1-5]. *C. elegans *is an attractive model system to study the unique and combined effects of *Hox *genes on developmental processes. However, our present knowledge on the roles of *Hox *genes in this organism is rather unbalanced as numerous studies have shed light into the mechanisms by which three *Hox *genes, *lin-39*, *mab-5 *and *egl-5*, control various developmental events, but there is almost no information on the function of the other *Hox *paralogs, *ceh-13*, *nob-1 *and *php-3*. In this study we describe novel roles for *ceh-13 *in different developmental processes, and show that its functional domain obvious along the anteroposterior body axis, representing a break in colinearity.

### *ceh-13 *influences Q cell migration

Our data presented here indicate that *ceh-13 *is required for the proper positioning of Q neuroblasts (Figs. 2, 3, 4). Inactivation of *ceh-13 *led to a shortened migration of the AVM (QR.paa) and PVM (QL.paa) cells. AVM was positioned improperly in nearly each of the *ceh-13 *deficient animals examined. Mislocalization of PVM was also obvious in a significant portion of *ceh-13 *mutant larvae. This latter cell migration defect is particularly intriguing as it manifests in the posterior body domain. Although *ceh-13 *is considered as an anterior (*labial*-like) *Hox *gene [14], its defect disrupted the ability of a QL descendant to migrate toward the posterior. Furthermore, elimination of *ceh-13 *function also caused mispositioining of ALM sensory neurons in the majority of *ceh-13 *mutants.

Upon these findings, it was relevant to ask whether *ceh-13 *interacts with *mig-13*, which affects AVM migration and whose expression is controlled by *mab-5 *[29]. In *ceh-13(-); mig-13(-) *double mutant animals, the migration of AVM was more severely affected than in *mig-13 *single mutants. Thus, the two genes may act parallel to control cell migration in the anterior body domain. Alternatively, *ceh-13 *influences both *mig-13 *activity and another pathway to affect this process. *mig-13 *expression was abolished upon *ceh-13 *deficiency, supporting the latter possibility. These data are consistent with previous findings reporting that *ceh-20 *is required for *mig-13 *activity to control cell migration in a *lin-39*-independent manner [25]. It is worth to note, however, that the regulatory region of *mig-13 *contains no canonical binding site for CEH-13. Thus, the effect of CEH-13 on *mig-13 *expression may be indirect. Together, we conclude that *ceh-13 *affects cell migration in body parts covered by functional domains of the middle *Hox *paralogs.

### *ceh-13 *affects the fusion of Pn.p cells in various body domains

The fusion of Pn.p cells with hyp7 at the early larval stages is known to be under the control of *lin-39 *and *mab-5 *[11,12]. According to our present data, *ceh-13 *is also involved in this process at least in hermaphrodites (Figure 5). Unexpectedly, *ceh-13*, unlike *lin-39*, appeared to promote Pn.p fusion, and this function was evident even in the posterior body part; besides the central P(3-8).p cells, additional Pn.p cells remained unfused in animals defective for CEH-13. These results indicate that *ceh-13 *opposes with *lin-39 *in certain biological processes. It is possible that the two *Hox *genes share common targets genes mediating cell fusion, but regulate them in an opposite way. Alternatively, *lin-39 *represses genes required for cell fusion, such as *eff-1 *[37], while *ceh-13 *promotes the expression of another set of genes that promote cell fusion. Nevertheless, *ceh-13 *and *lin-39 *may co-ordinately regulate cell fusion in certain Pn.p lineages. This can explain why the fusion pattern of Pn.p cells is quite different between *lin-39 *and *ceh-20 *mutant hermaphrodites [25]. Whereas in *lin-39 *lf mutant larvae almost all Pn.p cells fuse with hyp7, in *ceh-20(mu290) *mutants each VPC [P(3-8).p] and some of the most anterior and posterior Pn.p cells remain unfused [25].

### *ceh-13 *affects vulva patterning

*lin-39 *is required during the early larval stages for the P(3-8).p cells to remain unfused, and, later at the L3 larval stage, to generate vulval cell divisions [11,12]. Considering cell fusion, *ceh-13*, similarly to *ceh-20 *[25], appears to contrast with *lin-39*: it promotes, rather than prevents, fusion in Pn.p cells (see data above). The higher number of unfused Pn.p cells in *ceh-13 *lf mutants may be the reason of why these animals exhibited, although with a low penetrance, ectopic vulval induction (Figure 6). In controlling vulval cell division, however, *ceh-13 *acts along with *lin-39*; inactivation of *ceh-13 *reduced the ability of VPCs to undergo vulval induction in *synMuv AB *double mutant background (Figure 6). Consistently, *ceh-13 *is known to be expressed in the major hypodermal syncytium [13], in which *synMuv *genes antagonize Ras-mediated vulval induction [38]. It is also possible that *ceh-13 *indirectly affects vulval induction via modulating the expression of other *Hox *factors involved in vulval cell fate specification. Together, vulval patterning is another example in which the anterior *Hox *paralog *ceh-13 *acts in the middle body domain.

### Lethality of *ceh-13 *mutants can be suppressed by increased dosage of middle *Hox *paralogs

Double mutant animals defective for both *lin-39 *and *mab-5 *are viable and fertile. In contrast, the majority of *ceh-13(-) *single mutants arrest development at the embryonic or early larval stages [14]. In this study we show that extra copies of *lin-39 *or a gf mutation of *mab-5 *are able to rescue lethality in *ceh-13 *deficient animals. In addition, other aspects of the pleiotropic Ceh-13 phenotype, including malformations at various body parts, slow growth rate, reduced lifespan and lowered fertility, could also be restored to normal levels by *mab-5(e1751gf) *mutation (Figure 8). These results also indicate a functional redundancy between *ceh-13 *and the middle-group *Hox *paralogs in various body domains. As *ceh-13 *has an unusually wide expression and activity domain, extending from the head to tail, it may cover and substitute several functions of *lin-39 *and *mab-5*, making the middle *Hox *paralogs to be dispensable for development. In contrary, *lin-39 *and *mab-5 *operate in the middle and posterior body domains, respectively. Their site of action overlaps only partially with that of *ceh-13*. As a result, *ceh-13 *has evolved as a predominantly essential *Hox *gene.

## Conclusions

The nematode *Hox *genes are thought to have undergone rapid divergent evolution, probably due to the adaptation of the lineage-driven mode of development [7]. *ceh-13 *is the only anterior (*labial*-like) paralog encoded by the *C. elegans *genome. However, many of its characteristics make *ceh-13 *as a rather atypical anterior *Hox *gene. First, it is located between the two middle *Hox *paralogs, downstream of *lin-39 *and upstream of *mab-5*. This unusual genomic organization of *ceh-13 *resulted from an ancient inversion event, presumably soon after the emergence of *lin-39 *and *ceh-13 *from their common ancestor (Figure 8F). Second, the spatial expression domain of *ceh-13 *largely overlaps with that of the other *Hox *paralogs. Thus, its relative localization within the cluster and functional domain are not collinear with each other.

*ceh-13 *plays multiple roles in *C. elegans *development. It controls cell adhesion, tissue patterning in different parts of the body (i.e., morphogenesis), cell fusion, cell migration, growth rate and fertility [[14]; this study]. Some of these functions, such as cell adhesion and growth, might have characterized the primordial *Hox *gene that gave raise the ancestor *Hox *cluster through a series of tandem gene duplication and subsequent diversification events in an early phase of animal evolution. In this scenario, a *ceh-13*-like primordial *Hox *gene underwent gene duplication, resulting in an upstream and a downstream daughter that became the prototype of the first anterior paralog and the first posterior paralog, respectively. After a sufficient degree of diversification, these genes conferred anterior (head) and posterior (tail) polarizations for the host organism. The anterior paralog then underwent duplication again: the resulting downstream descendant evolved into the first middle *Hox *paralog, increasing moprhological complexity in the mid-body domain. The fact that a *ceh-13*-like ortholog presents in all nematode lineages examined so far supports this scenario [7]. The early inversion event that occurred between the ancestral anterior and middle *Hox *paralogs in the *C. elegans *lineage may conserved the broad expression domain and diverse developmental roles of the anterior descendant. Another possible, but much unlikely, interpretation for the unusual properties of *ceh-13 *is that its ancestor might have acquired various functions secondarily during evolution. The question, however, remains open why the other nematode *Hox *paralogs have avoided such evolutionary innovations.

## Methods

### Genetics and strains

Standard methods were used for culturing and manipulating *Caenorhabditis elegans *strains. Strains were raised at 20°C, unless indicated. Wild-type worms correspond to Bristol, strain N2 [39]. The following mutant and transgenic strains were used in this study:

*muIs35 *[MEC-7::GFP, *lin-15(+)*]*V*

*zhIs1 *[LIN-39::GFP, *unc-119(+)*]*IV; unc-119(e2498)III*

*swIs1 *[*ceh-13::gfp, rol-6(su1006)*]*II*

*muIs16 *[MAB-5::GFP, *dpy-20(+)*]*?; dpy-20(e1282)IV*

*bxls12 *[*egl-5::gfp*]

*jcIs1 *[*ajm-1::gfp*, *rol-6(su1006)*, *unc-29(+)*]*IV; unc-29(e193)I*

*muIs62 *[*pmig-13::gfp; lin-15(+)*]*?*

*mig-13(mu225)X; lin-15(n765ts)X; muIs62 *[*pmig-13::GFP, lin-15(+)*]

ceh-13(sw1)III

ceh-13(ok737)III

mab-5(e1751gf)III

*mab-5(e1751gf)III*; *ceh-13(sw1)III*

*mab-5(e1751gf)III; muIs35 *[MEC-7::GFP*; lin-15(+)*]*V*

*mab-5(e1751gf)III; ceh-13(sw1)III; muIs35 *[MEC-7::GFP*; lin-15(+)*]*V*

*mig-13(mu294)X; muIs35 *[MEC-7::GFP*; lin-15(+)*]*V*

*ceh-13(sw1)III; mig-13(mu294)X; muIs35 *[MEC-7::GFP*; lin-15(+)*]*V*

*ceh-13(sw1)III; muIs62 *[*pmig-13::gfp; lin-15(+)*]

*ceh-13(sw1)III; muIs35 *[MEC-7::GFP*; lin-15(+)*]*V*

*ceh-13(ok737)III; muIs35 *[MEC-7::GFP*; lin-15(+)*]*V*

let-60 (n1064gf)IV

lin-8(n111)II; lin-15B(n765ts)X

lin-53(n833)I; lin-15B(n765ts)X

ceh-13(sw1)III; let-60 (n1064gf)IV

ceh-13(sw1)III; lin-8(n111)II; lin-15B(n765ts)X

ceh-13(ok737)III; lin-8(n111)II; lin-15B(n765ts)X

ceh-13(sw1)III; lin-53(n833)I; lin-15B(n765ts)X

### Characterization of the mutation *ok737*

*ok737 *allele is a deletional derivative of *ceh-13*. It removes the end of the first exon and more than half of the first intron, resulting in a frame shift and the generation of a premature stop codon 26 base pairs after the downstream deletional breakpoint. Based on phenotypic characterization (see below), *ok737 *is likely to represent another null allele of *ceh-13*. Homozygous *ceh-13(ok737) *mutant animals exhibit a variable abnormal morphology phenotype accompanied with a highly penetrant embryonic or larval lethality: 96% (621/647) of *ok737 *mutants die at various stages of embryogenesis or at early larval stages. The arrested embryos and larvae display serious body malformations, especially at the anterior and central body parts. The *ceh-13(ok737) *escapers, which are able to develop into fertile adults, show less severe morphological defects than the arrested mutants, and have a decreased brood size (data not shown), a short body length and delayed developmental rate, as compared with the wild type. Homozygous *ceh-13(ok737) *mutants were isolated from the VC509 balanced strain of genotype *ceh-13(ok737) III/hT2*[*bli-4(e937) let-?(q782) qIs48*] *(I;III)*. For sequencing the mutant allele, genomic PCR was performed with the following forward and reverse primers: 5'-tga gct cca ctg aat gtt atg g-3' and 5'-tat gac gaa ccg gtc ttt cc-3'.

### Assaying the positions of Q descendants

The position of AVM (QR.paa) and PVM (QL.paa) was determined by an integrated *mec-7::gfp *reporter [28]. Their location was scored as described preciously [25,29]. Statistical analysis of cell distribution was performed by the software MATLAB. The position of PVM was also screened by using an integrated *tax-4::gfp *fusion reporter [29].

### Assaying cell fusion in Pn.p lineages

Fused vs. unfused fates of Pn.p cells were determined at the end of the L1 larval stage by using an integrated *ajm-1::gfp *reporter [30], which marks a component of adherent junction, thereby labeling the outline of unfused cells. Analysis of vulval patterning was performed by differential interference contrast (DIC) microscopy. Images were collected from an Olympus BX-51 microscope equipped with an F-WU II camera. Statistical analysis of vulva phenotypes was performed by unpaired *t*-test, using the software SPSS 14.0.

### Construction of *gfp *reporters

A 15 kb large genomic fragment consisting of 10 kb upstream regulatory element and almost the entire *nob-1 *coding region except for the last codon, was PCR amplified by using the following primers. Forward primer: 5'-aac tga gaa cca atg cat tgg ctc cta tca cgg ggt tct gg-3', reverse primer: 5'-cgg gat ccc ggt tga tca atc gct cga tgc-3'. The resulting fragment was digested with PstI and BamHI, and cloned into the expression vector pPD95.75. Transgenic lines were produced by microinjection, using the co-transformation marker *rol-6(su1006)*. Extrachromosomal transgenes then was integrated by UV radiation and subsequent isogenization by crossing back with the wild type 8 times. For amplifying the *php-3 *coding regions, genomic PCR was performed by the following forward and reverse primers: 5'-tgt ttc tca aaa acg gat gg-3' and 5'-cgg gat ccc gcg tag gca gtt gtg cag ctc ttg tc-3'. The PCR fragment was digested with MluI and BamHI, and cloned into the *nob-1::gfp*-containing vector (Note that *nob-1 *and *php-3 *are adjacent genes and use the same promoter region for regulating expression). Transgenic worms containing the extrachromosomal array were generated as described above.

### Lifespan assays

Lifespan assays were carried out at 25°C. For synchronization, 20-30 gravid well-fed adults (P) were transferred to a new agar plate containing nematode growth medium (NGM) seeded with *E. coli *OP50 to lay eggs for 4-5 hours, and then removed. F1 young (not gravid) adults were transferred to NGM plates supplemented with 300 mg/ml FUDR (5-fluoro-2'-deoxyuridine) for 1 day (t = 0). This treatment inhibited the germ-line to produce germ cells. Sterile F1 adults were then transferred to the final assay plates and scored. SPSS 14 software was used to calculate mean lifespan and perform statistical analysis. p values for comparing Kaplan-Meyer survival curves between two groups were determined using log-rank (Mantel-Cox) tests.

### Phylogenetic analysis

For clustering the *HOX *genes from *C. elegans *(AC numbers: *ceh-13*: NM_066254; *egl-5*: L19247; *lin-39*: L19248; *mab-5*: AF277990; *nob-1*: AF172090; *php-3*: AF172092), we applied the Bayesian phylogenetic method and used 180 nucleotide long conserved homedomain sequences. For calculating the tree, MrBayes v3.1.2 software was used. The *codon *likelihood model was applied with one substitution type and with invariable proportion of sites. Te rate for the remaining sites were drawn from an estimated gamma distribution. The number of generations was set 1.000.000 and two independent runs were done. Values on the tree correspond to the posterior probability - i.e. clade credibility - of each node [from 0 (minimum) to 1 (maximum)].

## Authors' contributions

BT made the majority of the experiments, wrote the manuscript, performed statistical analysis. T. V designed experiments, performed the expression analysis of the Hox genes, wrote the manuscript. Á R performed vulval analysis in *ceh-13 *and *synMuv *mutant animals. EA performed the phylogenetic analysis of the nematode *Hox *genes. FM designed experiments, wrote the manuscript. KTV supervised the work, designed experiments, performed cloning experiments, wrote the manuscript. All authors read and approved the final manuscript.
